# The association of time and medications with changes in bone mineral density in the 2 years after critical illness

**DOI:** 10.1186/s13054-017-1657-6

**Published:** 2017-03-21

**Authors:** Neil R. Orford, Michael Bailey, Rinaldo Bellomo, Julie A. Pasco, Claire Cattigan, Tania Elderkin, Sharon L. Brennan-Olsen, David J. Cooper, Mark A. Kotowicz

**Affiliations:** 1 Barwon Health, Geelong, VIC Australia; 2 Australian and New Zealand Intensive Care Research Centre (ANZIC-RC), Department of Epidemiology and Preventive Medicine (DEPM), Monash University, Melbourne, VIC Australia; 3 School of Medicine, Deakin University, Geelong, VIC Australia; 4 Department of Epidemiology and Preventive Medicine (DEPM), Monash University, Melbourne, VIC Australia; 5 Melbourne Medical School-Western Campus, Department of Medicine, The University of Melbourne, St Albans, VIC Australia; 6 Australian Institute for Musculoskeletal Science (AIMSS), The University of Melbourne, Melbourne, VIC Australia; 7 Institute for Health and Ageing, Australian Catholic University, Melbourne, VIC Australia; 8 Intensive Care Unit, University Hospital Geelong, Ryrie St, Geelong, VIC 3220 Australia

**Keywords:** Critical illness, Long-term outcomes, Osteoporosis, Fracture, Bone loss, Bone mineral density, Bone turnover marker

## Abstract

**Background:**

Critical illness is associated with increased risk of fragility fracture and loss of bone mineral density (BMD), although the impact of medication exposures (bone anti-fracture therapy or glucocorticoids) and time remain unexplored. The objective of this study was to describe the association of time after ICU admission, and post-ICU administration of bone anti-fracture therapy or glucocorticoids after critical illness, with change in BMD.

**Methods:**

In this prospective observational study, conducted in a tertiary hospital ICU, we studied adult patients requiring mechanical ventilation for at least 24 hours and measured BMD annually for 2 years after ICU discharge. We performed mixed linear modelling to describe the association of time, and post-ICU administration of anti-fracture therapy or glucocorticoids, with annualised change in BMD.

**Results:**

Ninety-two participants with a mean age of 63 (±15) years had at least one BMD assessment after ICU discharge. In women, a greater loss of spine BMD occurred in the first year after critical illness (year 1: -1.1 ± 2.0% vs year 2: 3.0 ± 1.7%, *p* = 0.02), and anti-fracture therapy use was associated with reduced loss of BMD (femur 3.1 ± 2.4% vs -2.8 ± 1.7%, *p* = 0.04, spine 5.1 ± 2.5% vs -3.2 ± 1.8%, *p* = 0.01). In men anti-fracture and glucocorticoid use were not associated with change in BMD, and a greater decrease in BMD occurred in the second year after critical illness (year 1: -0.9 ± 2.1% vs year 2: -2.5 ± 2.1%, *p* = 0.03).

**Conclusions:**

In women a greater loss of spine BMD was observed in the first year after critical illness, and anti-fracture therapy use was associated with an increase in BMD. In men BMD loss increased in the second year after critical illness. Anti-fracture therapy may be an effective intervention to prevent bone loss in women after critical illness.

**Electronic supplementary material:**

The online version of this article (doi:10.1186/s13054-017-1657-6) contains supplementary material, which is available to authorized users.

## Background

Over the last two decades the focus of research on intensive care outcomes has broadened from survival to include morbidity and quality of life [1–10]. To date, interventions aimed at improving recovery after critical illness have had a functional focus - physical therapy programs [11, 12], mental health support [13], and follow-up clinics [14–16] - but met with limited success [17]. However, there has been little focus on post-intensive care unit (ICU) bone loss, a potentially treatable condition.

In recent years, an association between critical illness and accelerated bone turnover has been described, including an increase in bone turnover markers (BTM) during critical illness [18], accelerated loss of bone mineral density (BMD) in the year following critical illness [19], and increased fragility fractures in survivors of critical illness [20]. This association was, as expected, most pronounced in older women [19, 20]. The annual change in femur and spine BMD in women that survived critical illness was -1.96% and -2.85%, compared to -0.65% and -0.18% in age-matched community controls [19]. The risk of fragility fracture for women greater than 60 years of age was significantly higher following critical illness than age-matched controls [20].

However the duration of this effect, and the potential impact of medications that are known to adversely (such as glucocorticoids [21, 22]) or positively (anti-fracture therapy) affect BMD and fracture risk, are not fully elucidated, and no long-term prospective studies have described the BMD outcome association after critical illness.

The aim of this study was to describe the association of time, post-ICU administration of bone anti-fracture therapy and glucocorticoids on change in BMD over a 2-year period in survivors of critical illness.

## Methods

### Design, ethics and consent

We conducted a prospective observational cohort study of longitudinal changes in BMD for a 2-year period after critical illness. Prior to commencement, approval was obtained from the Barwon Health Human Research Ethics Committee. Written informed consent was obtained from surrogate decision-makers and patients for inclusion for the first year of the study [19]. Subsequent consent was obtained from patients to extend follow-up to 2 years post critical illness.

### Study population

Adult (age greater than 20 years) patients admitted to a tertiary, mixed medical, surgical, and cardiac surgical ICU during the study period, and with duration of mechanical ventilation greater than 24 hours were eligible for enrolment in the study. Exclusion criteria included active malignancy, existing neurological illness with impaired weight bearing, inability to lie flat, metabolic bone disease, pregnancy, weight greater than 120 kilograms, and considered unlikely to survive by the treating intensivist. Patients with multiple ICU admissions during the study period were included for the first ICU admission only.

### Data collection

Data collected included demographics, osteoporosis risk factors [parental history of hip fracture, previous fragility fracture, body mass index (BMI) less than 20, current smoking, use of glucocorticoids, rheumatoid arthritis, alcohol consumption of three units daily or greater, or secondary causes of osteoporosis], information relating to critical illness and ICU interventions, ICU and hospital length of stay, survival, serum biochemistry, serum bone formation marker: type 1 N-terminal procollagen (P1NP), serum bone resorption marker: collagen type 1 cross-linked c-telopeptide (CTX), and BMD. BMD was measured by dual-energy X-ray absorptiometry (DXA) (Lunar; GE Healthcare, Madison, WI, USA), at the proximal femur (femoral neck) and lumbar spine. Short-term precision in vivo was 1.6% for the femoral neck and 0.6% for the lumbar spine. Details on the measurement of serum BTMs are provided in Additional file 1. Medication history included medications taken by participants prior to critical illness, during critical illness, and during follow-up periods. Use of anti-fracture therapy was defined as use of a bisphosphonate, strontium ranelate, teriparatide, denosumab, or raloxifene, in the previous year. Use of glucocorticoids was defined as greater than 3 months’ use in the previous year at a dose of prednisolone of 5 mg daily or more (or equivalent dose of other glucocorticoids).

Data were collected at ICU baseline (demographic data, clinical information, BTMs), post-ICU discharge (BMD), 1-year post-ICU discharge (BMD, BTMs, clinical information), and 2-year post-ICU discharge (BMD, clinical information). Details of the study operating procedure are provided in Additional file 2. BMD was presented as an absolute value (g/cm^2^), annualised percentage change (difference between BMDs divided by initial post-ICU discharge BMD calculated as an annualised rate), and categorised as normal (T-score > -1.0), osteopenic (T-score -2.5 to -1.0), or osteoporotic (T-score < -2.5). The T-score is the number of standard deviations above or below the young adult mean, based on WHO criteria [23] with cutoff values calculated from the Australian reference ranges [24, 25].

### Outcomes

The outcomes of the study were annualised percentage change compared to baseline BMD (lumbar spine and dual femoral neck) for each of the 2 years after ICU discharge. The effect of the post-ICU variables including year post-ICU discharge, anti-fracture therapy use, and glucocorticoid use, on annual percentage change in BMD were also assessed.

### Statistical analysis

All data were initially assessed for normality. Group comparisons were performed using chi-square tests for equal proportion, Student *t* tests for normally distributed data and Wilcoxon rank sum tests otherwise, with results reported as number (%), mean (standard deviation) or median (interquartile range) respectively. Mixed linear modelling was used to explore the nature of the relationship between anti-fracture therapy use, glucocorticoid use, and the mean annualised change in bone mineral density using all available data. Given the known differences in BMD between men and women, all results have been stratified by sex. To account for potential survival bias due to participant drop-out, additional sensitivity analysis was conducted considering only patients that completed all three BMD measurements over the 2-year study period. Finally, to further establish the duration and magnitude of change in BMD after critical illness in the absence of known modifiers, a final subgroup of completers excluding those with post-ICU glucocorticoid or anti-fracture therapy use was considered. All modelling results are reported as least square means ± standard errors and a two-sided *p* value of 0.05 was used to indicate statistical significance. Statistical analysis was performed using SAS version 9.4 (SAS Institute Inc., Cary, NC, USA), with figures produced using Graphpad Prism 7.0 © (GraphPad Software, San Diego, CA, USA).

## Results

### Patient enrolment

**Fig. 1:**
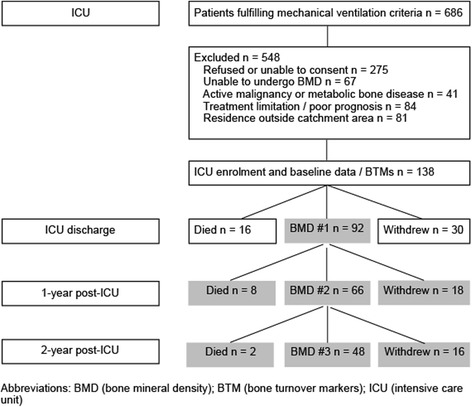
Summary of eligibility, enrolment, and follow-up of patients undergoing BMD assessment following intensive care. *Abbreviation*s: *BMD* bone mineral density, *BTM* bone turnover markers, *ICU* intensive care unit

A total of 92 of 138 patients enrolled in the study during their ICU stay underwent initial BMD assessment following ICU discharge and were eligible for this study. Of the 92 subjects, 66 had two BMD assessments, and 48 had all three BMD assessments over the 2-year study period (Fig. 1).

### Baseline characteristics

Baseline characteristics are presented in Table 1 stratified by BMD assessment status. Overall 40 (44%) of participants had at least one osteoporosis risk factor, 29 (32%) received glucocorticoids during critical illness, median ICU length of stay was 6 days [IQR 4,9], and hospital length of stay was 16 days [IQR 11,30]. Mortality at 1 year was 9%, and at 2 years was 11%. The 48 participants that completed all BMD assessments and were included in the sensitivity analysis were compared to the 44 participants that withdrew or died prior to completion of all BMD assessments. The groups had similar characteristics, except for an increased prevalence of osteoporosis risk factors (57% vs 31%, *p* = 0.02) in the group that withdrew or died prior to completion of all assessments.

**Table 1:** Demographic, clinical characteristics, baseline bone turnover markers, biochemistry, and outcomes by bone mineral density assessments at 2-year follow-up

Variable	All (*n* = 92)	Completed three BMD assessments (*n* = 48)	Completed one to two BMD assessments (*n* = 44)	*p* value
Age (yrs)	63.4 (±14.7)	65.8 (±11.4)	60.8 (±17.4)	0.1
BMI	27.1 (±5.1)	27.2 (4.4)	27.1 (5.8)	0.9
Women	44 (47.8)	22 (45.8)	22 (50.0)	0.8
Any osteoporosis risk factor	40 (43.5)	15 (31.3)	25 (56.8)	0.02
Co-morbidity				
Renal	7 (7.6)	4 (8.3)	3 (6.8)	1.0
Cardiovascular	40 (43.5)	22 (45.8)	18 (40.9)	0.7
Respiratory	22 (23.9)	8 (16.7)	14 (31.8)	0.1
APACHE III score	74.4 (±29.5)	76.3 (±29.3)	72.4 (±30.0)	0.5
ICU admission category				
Medical	54 (58.7)	26 (54.2)	28 (63.6)	
Cardiothoracic surgery	16 (17.4)	10 (20.8)	6 (13.6)	
General surgery	22 (23.9)	12 (25.0)	10 (22.7)	
ICU biochemistry and biomarkers				
Albumin (g/L)	23.9 (±5.8)	23.6 (±5.9)	24.1 (±5.7)	0.6
Calcium adj (mmol/L)	1.99 (±0.33)	2.01 (±0.32)	1.96 (±0.33)	0.5
Creatinine (umol/L)	116 [85, 178]	125 [85, 175]	109 [89, 196]	0.7
Vitamin D (nmol/L)	44.2 (±20.2)	43.4 (±20.0)	45.1 (±20.7)	0.7
Phosphate (mmol/L)	0.70 [0.52, 1.00]	0.67 [0.49, 1.04]	0.72 [0.52, 0.96]	0.7
PTH (pmol/L)	9.29 (±7.51)	9.88 (±7.94)	8.65 (±7.05)	0.4
CTX (ng/L)	581 [400, 851]	581 [386, 884]	581 [414, 837]	1.0
P1NP (ug/L)	31.5 [22.0, 60.0]	30.5 [22.0, 46.0]	32.5 [22.5, 87.0]	0.2
Hospital interventions/outcomes				
Ventilation duration (hrs)	86.0 [47.4, 146.0]	80.4 [43.9, 118.0]	91.9 [52.3, 215.0]	0.1
Glucocorticoid	29 (31.5)	16 (33.3)	13 (29.5)	0.8
CRRT	15 (16.3)	6 (12.5)	9 (20.5)	0.3
ICU LOS (days)	6 [4, 9]	7 [4, 8]	6 [4, 11]	0.9
Hospital LOS (days)	16 [11, 30]	15 [11, 28]	17 [10, 32]	0.7
Baseline BMD (post-ICU discharge)				
ICU admit to BMD (days)	33 [13,58]	36 [14, 63]	33 [12,56]	
T-score femur	-0.8 (±1.5)	-1.0 (±1.4)	-0.7 (±1.5)	0.3
Absolute femur (g/cm^3^)	0.956 (±0.197)	0.941 (±0.183)	0.974 (±0.212)	0.4
T-score AP spine	0.1 [-1.4, 1.0]	-0.1 [-1.6, 0.8]	0.1 [-1.3, 1.2]	0.4
Absolute AP spine (g/cm^3^)	1.207 (±0.242)	1.200 (±0.228)	1.210 (±0.261)	0.7
Mortality				
1-year	8 (8.7)	0 (0)	8 (18.2)	
2-year	10 (10.9)	0 (0)	10 (22.7)	

Data are shown as mean (±standard deviation), median [interquartile range] or number (%). Complete BMD follow-up defined as all three post-ICU measurements performed during the 2-year period. Incomplete BMD follow-up defined as one or two post-ICU measurements performed. Reference ranges: vitamin D (<25 nmol/L = deficient, 25–50 nmol/L insufficient, >50 nmol/L sufficient), PTH (range 1.6–6.9 pmol)

*Abbreviation*s: *BMD* bone mineral density, *BMI* body mass index, *APACHE* Acute Physiology and Chronic Health Evaluation, *ICU* intensive care unit, *PTH* parathyroid hormone, *CTX* collagen type 1 cross-linked c-telopeptide, *P1NP* type 1 N-terminal procollagen, *CRRT* continuous renal replacement therapy, *LOS* length of stay, *AP* anterioposterior

### Change in BMD and association with time, anti-fracture therapy and glucocorticoids

Over the 2-year post-ICU period 92 participants underwent a total of 114 measurements of annual change in BMD (post-ICU year 1 *n* = 66, post-ICU year 2 *n* = 48) (Table 2). Over the 2-year period ten participants were prescribed anti-fracture therapies (six women, four men), including alendronate (five participants), denosumab (two participants), strontium ranelate (two participants), and risedronate (one participant). Three (10%) women and one (3%) man received anti-fracture therapies in year 1 post-ICU, and six (27%) women and four (15%) men received anti-fracture therapies in year 2. Glucocorticoids were received by two (7%) women and one (2%) man in year 1 post-ICU, and five (23%) women in year 2.

**Table 2:** Bone mineral density assessments performed and results for entire cohort by gender

	All (*n* = 92^a^)			Women (*n* = 44^a^)			Men (*n* = 48^a^)		
Variable	Baseline	1 year	2 years	Baseline	1 year	2 years	Baseline	1 year	2 years
BMD studies performed	92	66	48	44	31	22	48	35	26
Anti-fracture therapy in prior year	-	4 (6.1)	10 (20.8)	-	3 (9.7)	6 (27.3)	-	1 (2.9)	4 (15.4)
Glucocorticoid in prior year	-	3 (4.5)	5 (10.4)	-	2 (6.5)	5 (22.7)	-	1 (2.9)	0 (0)
BMD measurement									
Femur T-score	- 0.8 (±1.5)	-1.0 (±1.4)	- 1.1 (±1.3)	- 1.2 (±1.4)	- 1.3 (±1.2)	- 1.4 (±0.9)	- 0.5 (±1.4)	- 0.7 (±1.5)	- 1.0 (±1.6)
Femur absolute (g/cm^3^)	0.956 (±0.197)	0.940 (±0.193)	0.923 (±0.178)	0.876 (±0.176)	0.872 (±0.161)	0.871 (±0.126)	1.028 (±0.187)	0.999 (±0.202)	0.964 (±0.203)
AP spine T-score	- 0.2 (±1.9)	- 0.2 (±1.9)	- 0.1 (±1.9)	- 0.7 (±1.8)	- 0.6 (±1.6)	- 0.5 (±1.2)	0.3 (±1.8)	0.2 (±2.0)	0.3 (±2.2)
AP spine absolute (g/cm^3^)	1.207 (±0.242)	1.205 (±0.241)	1.211 (±0.231)	1.135 (±0.250)	1.142 (±0.223)	1.151 (±0.173)	1.273 (±0.217)	1.260 (±0.246)	1.262 (±0.264)

Data are shown as mean (±standard deviation) or number (%)

*Abbreviations*: *BMD* bone mineral density, *AP* anterioposterior

^a^At baseline femur BMD not measured in three participants (one woman, two men). At 1 year femur BMD not measured in two participants (one woman, one man), at 2 years femur BMD not measured in one participant (one woman)

**Fig. 2:**
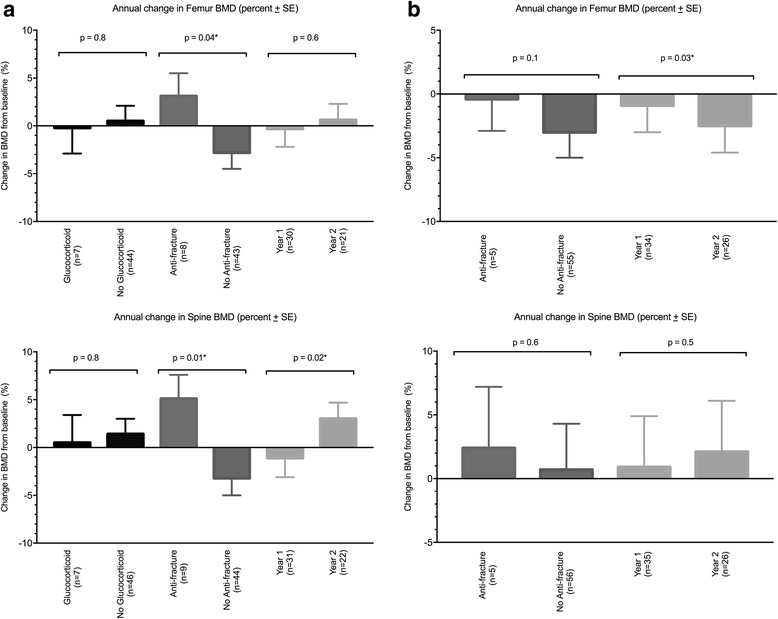
**a** RMANOVA assessment of annual BMD change in women. *Abbreviations*: *BMD* bone mineral density, *RMANOVA* repeat measure analysis of variance, *SE* standard error. **b** *RMANOVA* assessment of annual BMD change in men. *Abbreviation*s: *BMD* bone mineral density, *RMANOVA* repeat measure analysis of variance, *SE* standard error

In 44 women with 53 measurements of annual change in BMD over the 2-year period, a significantly greater decrease in BMD was observed in post-ICU year 1 compared to year 2 for spine BMD (year 1: -1.1 ± 2.0% vs year 2: 3.0 ± 1.7%, *p* = 0.02), but not femur BMD (year 1: -0.3 ± 1.9% vs year 2: 0.6 ± 1.7%, *p* = 0.6) (Fig. 2a). The use of anti-fracture therapy associated with a significant difference in post-ICU annual change of BMD, with an increase in BMD in participants who received anti-fracture medication compared to a decrease in those that did not (femur 3.1 ± 2.4% vs -2.8 ± 1.7%, *p* = 0.04, spine 5.1 ± 2.5% vs -3.2 ± 1.8%, *p* = 0.01). In women use of glucocorticoids was not associated with a difference in annual change in BMD compared to no use (femur -0.2 ± 2.7% vs 0.5 ± 1.6%, *p* = 0.8, spine 0.5 ± 2.9% vs 1.4 ± 1.6%, *p* = 0.8).

In 48 men with 61 measurements of annual change in BMD over the 2-year period, a greater annual decrease in femur BMD was observed in post-ICU year 2 compared to year 1 (year 1: -0.9 ± 2.1% vs year 2: -2.5 ± 2.1%, *p* = 0.03), with no difference in annual change of spine BMD (year 1: 0.9 ± 4.0% vs year 2: 2.1 ± 4.0%, *p* = 0.45). In men no association between anti-fracture therapy use and annual change in BMD was observed (femur -0.4 ± 2.5% vs -3.0 ± 2.0%, *p* = 0.1, spine 2.4 ± 4.8% vs 0.7 ± 3.6%, *p* = 0.6) (Fig. 2b). As only one male participant received glucocorticoids, analysis was not performed.

The sensitivity analysis for the 48 participants who completed all three BMD assessments is presented in Additional files 3 and 4. For women the percentage of participants with osteoporosis or osteopenia was 59% at ICU discharge, 68% at year 1, and 59% at year 2. In men the proportion was 39% at ICU discharge, 50% at year 1, and 54% at year 2. The results of sensitivity analysis for this group are presented in Additional file 4, and are consistent with the primary analysis.

### Annual change in BMD in participants not receiving glucocorticoids or anti-fracture therapy

**Fig. 3:**
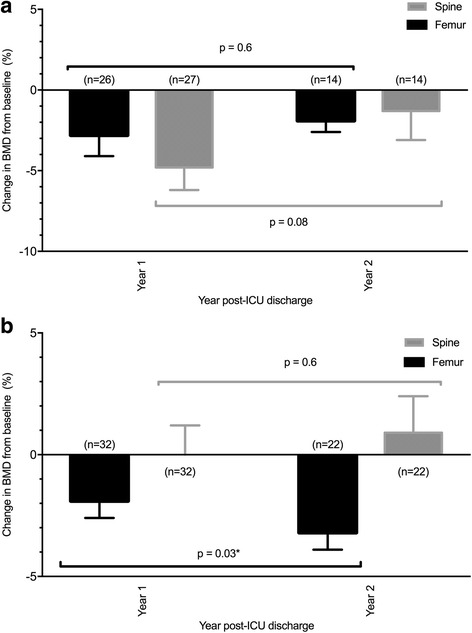
**a** Annual change in BMD in women not receiving anti-fracture or corticosteroid medications. **b** Annual change in BMD in men not receiving anti-fracture or corticosteroid medications. *Abbreviations*: *BMD* bone mineral density, *ICU* intensive care unit, *SE* standard error

The annual change in BMD in the first and second years after ICU discharge in the cohort of participants who did not receive either glucocorticoids or anti-fracture therapies are presented by sex in Fig. 3a and b. In women an annual decrease in femur and spine BMD was observed for both year 1 and 2, with no significant change over the 2-year period (femur -2.8 ± 1.3% vs -1.9 ± 0.7, *p* = 0.6, spine -4.8 ± 1.4% vs -1.3 ± 1.8%, *p* = 0.08). In men the annual decrease in femur BMD was significantly greater in year 2 than year 1 (femur -1.9 ± 0.7% vs -3.2 ± 0.7%, *p* = 0.03), with no difference in annual spine BMD change between year 1 and year 2 (spine 0.0 ± 1.2% vs 0.9 ± 1.5%, *p* = 0.6).

## Discussion

### Key findings

We studied the association between time, post-ICU administration of bone anti-fracture therapy and glucocorticoids, and annual change in BMD over a 2-year period after critical illness. In women a significantly greater loss of spine BMD was observed in the first year after ICU compared to the second. In women who did not receive anti-fracture therapy or glucocorticoids, a decrease in BMD was observed in both years after ICU discharge. However, post-ICU administration of anti-fracture therapy was associated with an increase in BMD, compared to a decrease in women who did not. In men, loss of femur BMD was significantly greater in the second year after ICU discharge. There was no association between use of anti-fracture therapy or glucocorticoids and change in BMD, although only a small number of men received post-ICU treatment.

### Relationship to previous studies

Loss in BMD following critical illness has been reported in two previous studies. A significant decrease in calcaneal BMD was observed over 10 days in patients with acute respiratory distress syndrome [26], although this result is limited by precision error of portable BMD devices and short time frame [27]. We described a significant decrease in spine and femur BMD in the first year after ICU admission, greater than age- and gender-matched community controls, in the initial cohort from this study [19]. In addition a number of studies have described abnormal BTMs during and after critical illness, of a magnitude similar to that described in postmenopausal women’s or metabolic bone disease [19, 28–34]. High bone turnover and bone loss, due to negative remodelling balance at the basic multicellular unit, has been described as an independent risk factor for fracture [29, 35]. An increased fracture risk in older women after intensive care compared to matched population controls has been described [20].

The extension of BMD assessment to 2 years after critical illness in this study adds important information about the time course and magnitude of changes in BMD following critical illness [19, 26]. In women we observed a loss in femur and spine BMD in the first 2 years after critical illness, with recovery of BMD observed in women receiving anti-fracture therapy. The reported change in BTMs after critical illness describes increased resorption markers during and after ICU [19, 28–34], followed by increased formation markers and normalisation of resorption markers by 1 year [19]. The magnitude of this decrease was greater than we have previously observed in community controls [19], supporting the hypothesis that factors associated with critical illness contribute to an increase in bone loss, and that administration of anti-fracture therapy is a major determinant of BMD recovery after critical illness. The different pattern of BMD loss in men compared to women following critical illness is also of interest. The observed decrease in femur BMD is consistent with our previous study of change in BMD after ICU compared to community controls [19]. The significantly greater loss in femur BMD in the second year after ICU discharge, the high proportion of men with osteoporosis and osteopenia at 2 years post-ICU discharge, and the low rate of post-ICU anti-fracture treatment, suggest further investigation of risk factors and consequences of bone loss in men is warranted.

The current literature regarding the relationship between anti-fracture therapy use and change in BMD following critical illness is limited. A small study reported a transient decrease in bone resorption markers after administration of intravenous ibandronate [36], and a retrospective propensity-matched cohort study described an association between pre-ICU bisphosphonate use and reduced mortality [37]. In addition, serial computed tomography (CT) assessment of vertebral BMD revealed bisphosphonate users had lower baseline bone density and an attenuated decrease in BMD during critical illness. This study is the first to prospectively describe an association between anti-fracture therapy use and change in BMD over a prolonged period following critical illness. The observed increased proportion of anti-fracture therapy use in women is expected, based on lower measured BMDs in the years after critical illness. The observed positive association between anti-fracture therapy use and BMD provides support for future interventional studies in this population.

The observation that use of glucocorticoid, a known risk factor for osteoporosis, was not associated with an increase in annual change in BMD was interesting, although limited by small sample size and the risk of type II error. More prospective data on the relationship between BMD changes following critical illness and the effect of known osteoporosis factors, including medications administered before and after critical illness, are required to further elucidate these relationships.

### Study implications

This study implies that critical illness is associated with prolonged and sustained loss of BMD, with variable effects on femur and spine in women and men. Although recovery of BMD occurs overall in women, this may be associated with the use of anti-fracture therapy in the post-ICU period. This implies that anti-resorptive therapy may be an effective intervention to prevent bone loss in women with critical illness as has been shown in other at-risk patients.

### Strengths and limitations

Our study has several strengths. It is the first study to collect prospective data on bone density using DXA, the gold standard for BMD assessment, over a 2-year period after critical illness. This is important because the previously described changes in bone mineral density that occur immediately after critical illness may be attenuated over time. Moreover, understanding of the natural history of these changes can be used to guide the need and design of interventional trials. In addition, the collection of post critical illness medication history allows assessment of factors that are known to modify bone turnover, over a time frame required to assess this effect.

There are limitations to this study. The loss of a large proportion of patients prior to the 2-year follow-up due to death or withdrawal introduces limitations due to small sample size, including ability to assess the impact of multiple risk factors on post critical illness change in BMD, perform subgroup analysis, and introduces the possibility of type II error. However, the ability to assess the effect of anti-fracture therapy and glucocorticoids, although limited by numbers, provides unique and valuable information about feasibility and design of an interventional study. Also, the assessment of glucocorticoid use following critical illness was defined as use for greater than 3 months in the previous year, and it is possible that shorter duration of glucocorticoids during critical illness or recovery were associated with a change in BMD that was not captured. However, glucocorticoids are a known risk factor for loss of BMD, and a much larger study would be required to assess the effect of glucocorticoids administered before, during, and after ICU. Also, data relating to a number of variables associated with BMD was not collected, including other medications that affect bone turnover, nutrition, falls, and fractures. However, given the small sample size, analysis of the relationship between these factors and BMD would not have been possible. Finally, anti-fracture medications were clinician-initiated rather than randomised, introducing selection bias into the results. However, anti-fracture therapies are initiated in the highest risk patients with the lowest BMD, with the effect observed in this study likely to underestimate that observed in a mixed population of critically ill patients.

## Conclusions

We performed a prospective observational study of changes in BMD in critically ill, mechanically ventilated subjects, and observed a high prevalence of osteopenia and osteoporosis at 2 years post-ICU discharge. In women participants, a greater loss of spine BMD was observed in the first year after critical illness, with anti-fracture therapy use associated with an increase in BMD compared to a decrease in BMD in those that did not receive such therapy. In men BMD loss increased in the second year after critical illness, and there was no association between use of anti-fracture therapy or glucocorticoids and change in BMD, although only a small proportion of men received post-ICU bone-related medications. These findings suggest anti-fracture therapy may be an effective intervention to prevent bone loss in women with critical illness, and prospective trials investigating this effect are warranted.

## Additional files

Additional file 1: Measurement of bone turnover markers. Details of BTMs measurement. (DOC 22 kb)

Additional file 2: Study operating procedures. Details of study procedure and data collection time points from enrolment to completion. (DOCX 12 kb)

Additional file 3: Bone mineral density and T-score for the 2 years after critical illness in participants that completed all bone mineral density assessments. Bone mineral density and T-score at enrolment, 1 year, and 2 years after critical illness, presented overall and stratified by gender, for the 47 participants who completed all assessments. (DOCX 13 kb)

Additional file 4: Sensitivity analysis of annual BMD change in women and men. The sensitivity analysis of annual change in BMD compared to baseline for women and men who completed all three BMD assessments, with repeat measure analysis of variance to explore the relationship between anti-fracture use, glucocorticoid use, and time after ICU discharge. (DOCX 666 kb)

## Abbreviations

AP: Anterioposterior
APACHE: Acute Physiology and Chronic Health Evaluation
BMD: Bone mineral density
BMI: Body mass index
BTM: Bone turnover marker
CRRT: Continuous renal replacement therapy
CT: Computed tomography
CTX: Collagen type 1 cross-linked c-telopeptide
DXA: Dual-energy X-ray absorptiometry
ICU: Intensive care unit
LOS: Length of stay
P1NP: Type 1 N-terminal procollagen
PTH: Parathyroid hormone
