# Supplementary material for: The association of time and medications with changes in bone mineral density in the 2 years after critical illness
Source: Crit Care. 2017 Mar 21;21:69. doi: 10.1186/s13054-017-1657-6 (PMC5361814; doi:10.1186/s13054-017-1657-6)
Supplement: Supplementary file 1 — Measurement of bone turnover markers. Details of BTMs measurement. (DOC 22 kb) [file 13054_2017_1657_MOESM1_ESM.doc]

Additional File 1: Measurement of Bone Turnover Markers

The serum bone turnover markers CTX and P1NP were collected the morning after enrolment with routine early morning blood tests, and measured using the automated Roche Modular Analytics E170 analyser. Serum CTX limit of detection was 10 ng/L with inter-assay coefficient of variations (CVs) of 6.5% at 361 ng/L, 3.8% at 816 ng/L and 3.4% at 3304 ng/L (n = 10). Serum P1NP inter-assay CVs were 4.9% at 73 μg/L, 2.6% at 392 μg/L, and 2.1% at 768 μg/L (n = 10) with a limit of detection of 5μg/L.
