# Supplementary material for: The association of time and medications with changes in bone mineral density in the 2 years after critical illness
Source: Crit Care. 2017 Mar 21;21:69. doi: 10.1186/s13054-017-1657-6 (PMC5361814; doi:10.1186/s13054-017-1657-6)
Supplement: Supplementary file 2 — Study operating procedures. Details of study procedure and data collection time points from enrolment to completion. (DOCX 12 kb) [file 13054_2017_1657_MOESM2_ESM.docx]

Additional File 2: Study Operating Procedures

| Softer Study Procedures | |
| --- | --- |
| >24 hrs to <168 hrs duration of mechanical ventilation | |
| Enrolment | Inclusion criteria met, consent obtained |
| Study procedures | Baseline and demographic data |
|  | Biochemistry and BTM (serum PINP, CTx, Vit D, PTH, albumin, calcium, phosphate, creatinine) |
| ICU discharge (ICU discharge to 1-month) | |
| Study procedure | BMD #1 |
| 1 year follow-up (1 year post-ICU discharge) | |
| Study procedure | Contact participant |
|  | BMD #2 |
|  | Biochemistry and BTMs (serum PINP, CTx, vitamin D, PTH, albumin, calcium, phosphate, creatinine) |
|  | Medication history |
| 2 year follow-up (2 year post-ICU discharge) | |
| Study procedure | Contact participant |
|  | BMD #3 |
|  | Medication history |
| Vitamin D / calcium / anti-resorptive therapy will be offered to participants in accordance with current guidelines and review of results and risk factors by an endocrinologist | |

1. Abbreviations: BMD (bone mineral density), AP (anteroposterior)
