# Supplementary material for: The association of time and medications with changes in bone mineral density in the 2 years after critical illness
Source: Crit Care. 2017 Mar 21;21:69. doi: 10.1186/s13054-017-1657-6 (PMC5361814; doi:10.1186/s13054-017-1657-6)
Supplement: Supplementary file 3 — Bone mineral density and T-score for the 2 years after critical illness in participants that completed all bone mineral density assessments. Bone mineral density and T-score at enrolment, 1 year, and 2 years after critical illness, presented overall and stratified by gender, for the 47 participants who completed all assessments. (DOCX 13 kb) [file 13054_2017_1657_MOESM3_ESM.docx]

Additional File 3: Bone mineral density and T-score for the 2-years after critical illness in participants that completed all Bone Mineral Density Assessments.

| Variable | Baseline | 1-year | 2-year | P-value |
| --- | --- | --- | --- | --- |
| **All (n=48)** |  |  |  |  |
| BMD (g/cm2) |  |  |  |  |
| Dual Femur * | 0.941 (+0.183) | 0.922 (+0.181) | 0.923 (+0.178) | 0.006 |
| AP Spine | 1.200 (+0.228) | 1.182 (+0.242) | 1.211 (+0.231) | 0.04 |
| T score |  |  |  |  |
| Osteoporosis / osteopenia | 23 (47.9) | 28 (53.1) | 27 (56.3) |  |
| Normal | 25 (52.1) | 20 (41.7) | 21 (43.8) |  |
| **Women (n=22)*** |  |  |  |  |
| BMD (g/cm2) |  |  |  |  |
| Dual Femur* | 0.880 (+0.143) | 0.862 (+0.135) | 0.871 (+0.126) | 0.4 |
| AP Spine | 1.146 (+0.193) | 1.100 (+0.190) | 1.151 (+0.173) | 0.006 |
| T score |  |  |  |  |
| Osteoporosis / osteopenia | 13 (59.1) | 15 (68.2) | 13 (59.1) | 0.77 |
| Normal | 9 (40.9) | 7 (31.8) | 9 (40.9) |  |
| **Men (n=26)** |  |  |  |  |
| BMD (g/cm2) |  |  |  |  |
| Dual Femur | 0.990 (+0.198) | 0.971 (+0.201) | 0.964 (+0.203) | 0.0002 |
| AP Spine | 1.247 (+0.248) | 1.251 (+0.263) | 1.262 (+0.264) | 0.6 |
| T score femur |  |  |  |  |
| Osteoporosis / osteopenia | 10 (38.5) | 13 (50.0) | 14 (53.8) | 0.5 |
| Normal | 16 (61.5) | 13 (50.0) | 12 (46.2) |  |

Data are shown as mean (+standard deviation) or number (%)

Abbreviations: BMD (bone mineral density), AP (anteroposterior)

* At baseline femur BMD not measured in 1 woman, at 1-year femur BMD not measured in 1 woman, at 2-year femur BMD not measured in 1 woman.
