# Supplementary material for: The association of time and medications with changes in bone mineral density in the 2 years after critical illness
Source: Crit Care. 2017 Mar 21;21:69. doi: 10.1186/s13054-017-1657-6 (PMC5361814; doi:10.1186/s13054-017-1657-6)
Supplement: Supplementary file 4 — Sensitivity analysis of annual BMD change in women and men. The sensitivity analysis of annual change in BMD compared to baseline for women and men who completed all three BMD assessments, with repeat measure analysis of variance to explore the relationship between anti-fracture use, glucocorticoid use, and time after ICU discharge. (DOCX 666 kb) [file 13054_2017_1657_MOESM4_ESM.docx]

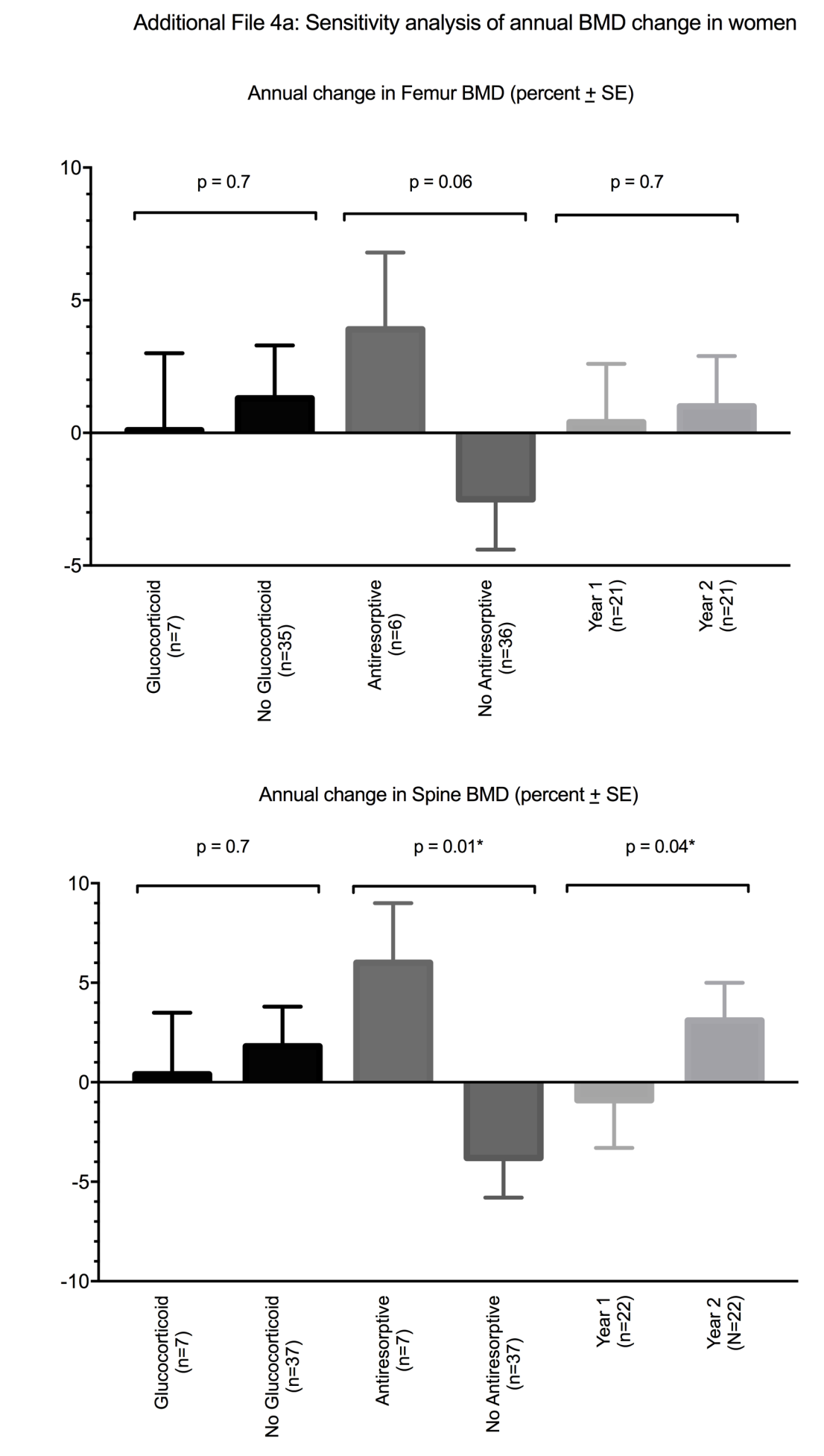


Abbreviations: BMD (bone mineral density); RMANOVA (repeat measure analysis of variance); SE (standard error)


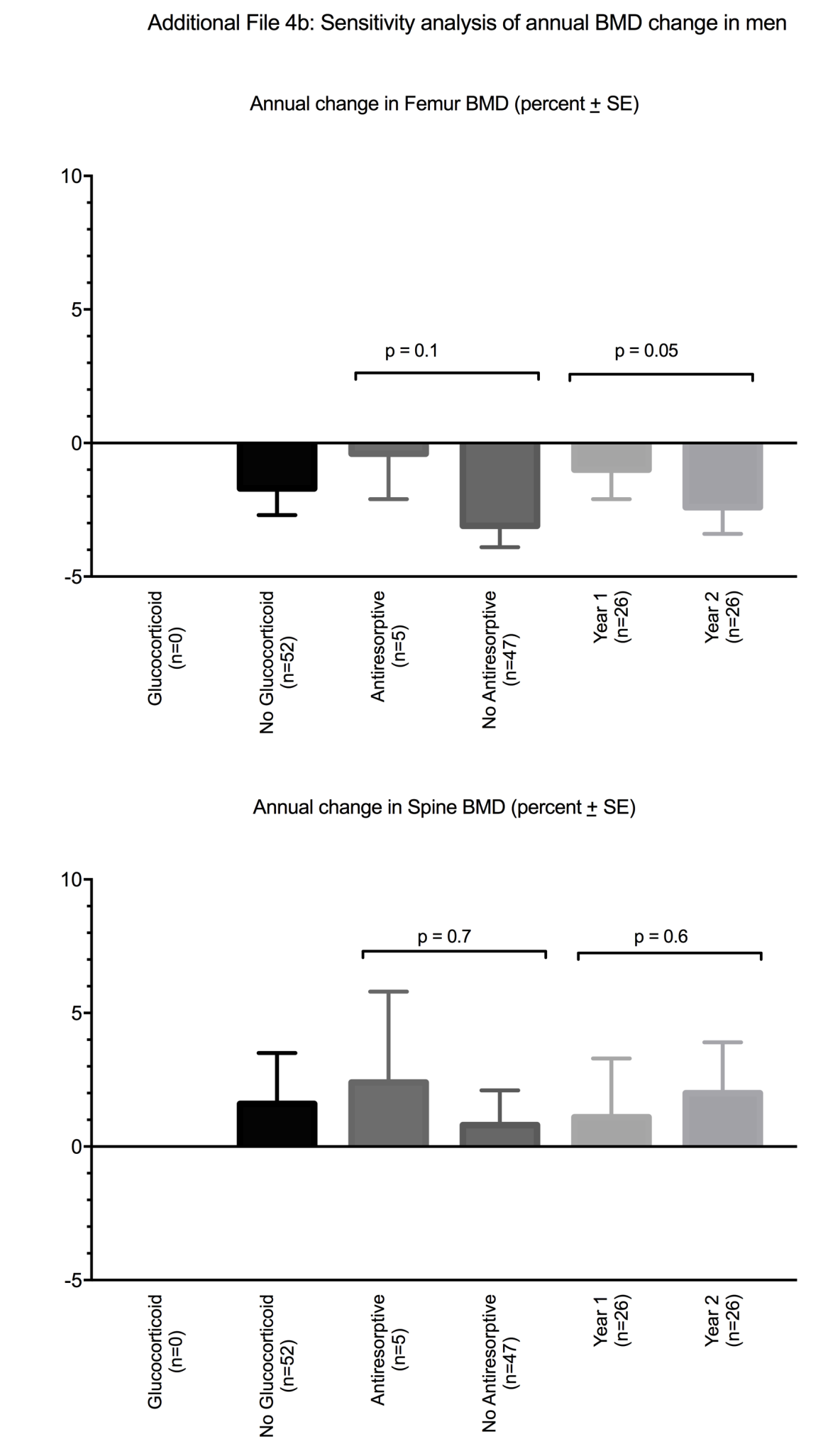
Abbreviations: BMD (bone mineral density); RMANOVA (repeat measure analysis of variance); SE (standard error)
